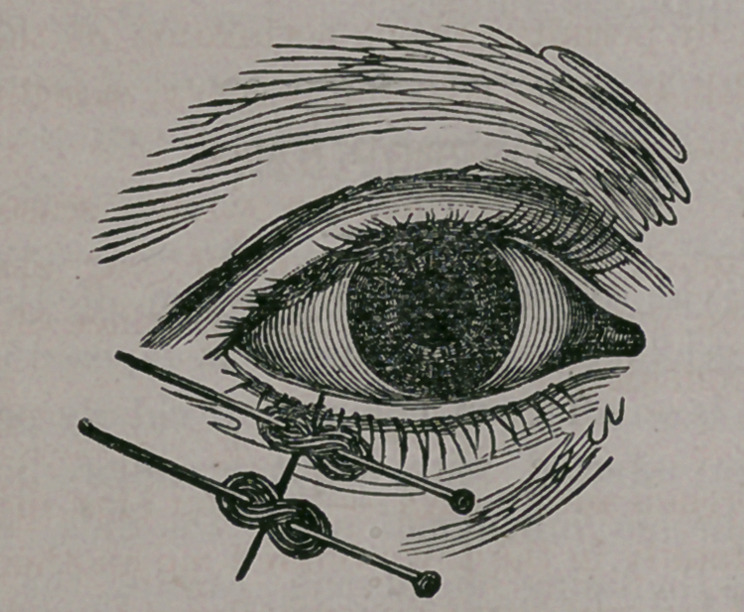# Ectropium

**Published:** 1877-10

**Authors:** 


### ECTROPIUM.

This is an out-turning or eversion of the eye lids, in which the mucus surface is exposed to the air. It is an affection common to elderly people, who are affected with slight granular lids or some chronic inflammation of the lids, which thickens the lining membrane, causing the eversion. When the delicate *conjunctiva* is thus exposed to the air, it becomes more and more thickened and inflamed; increasing the out-turning of the lid, until a ghastly deformity exists, giving the patient constant pain and inconvenience. The tears are turned from their natural channel and are found streaming over the cheek, irritating and excoriating the skin, while the eyes are literally swimming in the secretion.

Often, *ectropium* is caused by injuries, such as burns, scalds, or cuts near the eye, in the healing of which, the skin is contracted on the outside, producing the eversion. But, from whatever cause it occurs, the difficulty should be early remedied, before the resultant inflammation attacks the eye itself, so increasing the evil, which may lead to complete loss of the eye.

That the reader may the more readily comprehend how a deformity of this character is at once remedied by the skillful surgeon, we introduce two cuts illustrative of the procedure.

The first one shows the everted lid, with the lines indicating the direction of the surgeon's knife, in operating. A portion of the lid, included within the lines, is entirely removed, shortening the length of the lid by just the width of the excised portion, when the edges are again united. For this purpose, two delicate pins are thrust through the margin of the wound, the parts brought together by winding silk over the protruding ends, to bring them in apposition and at once correcting the deformity, as seen below:

Where the trouble exists from some injury shortening the skin upon the outside of the lids, or where a scar contracts the integument near the lids, so producing the deformity, the surgeon frequently inserts a piece of skin, allowing the parts to assume their natural position. But, from whatever cause it may occur, the remedy lies with the surgeon. Apply to him and be promptly relieved.

## Figures and Tables

**Figure f1:**
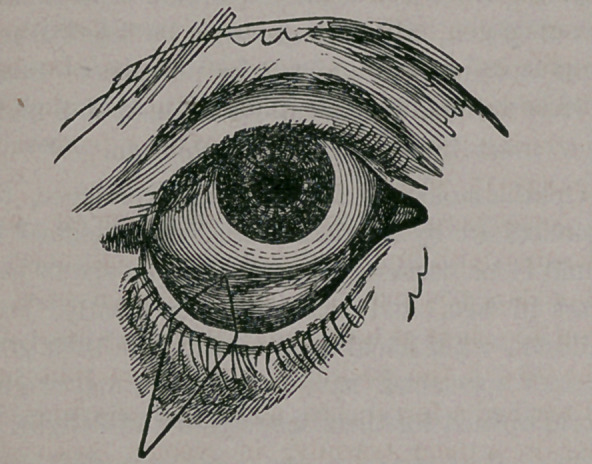


**Figure f2:**